# The Effect of TIP on Pneumovirus-Induced Pulmonary Edema in Mice

**DOI:** 10.1371/journal.pone.0102749

**Published:** 2014-07-21

**Authors:** Elske van den Berg, Reinout A. Bem, Albert P. Bos, Rene Lutter, Job B. M. van Woensel

**Affiliations:** 1 Pediatric Intensive Care Unit, Emma Children's Hospital, Academic Medical Center, Amsterdam, The Netherlands; 2 Department of Respiratory Medicine and Experimental Immunology, Academic Medical Center, Amsterdam, The Netherlands; The Ohio State University, United States of America

## Abstract

**Background:**

Pulmonary edema plays a pivotal role in the pathophysiology of respiratory syncytial virus (RSV)-induced respiratory failure. In this study we determined whether treatment with TIP (AP301), a synthetic cyclic peptide that mimics the lectin-like domain of human TNF, decreases pulmonary edema in a mouse model of severe human RSV infection. TIP is currently undergoing clinical trials as a therapy for pulmonary permeability edema and has been shown to decrease pulmonary edema in different lung injury models.

**Methods:**

C57BL/6 mice were infected with pneumonia virus of mice (PVM) and received TIP or saline (control group) by intratracheal instillation on day five (early administration) or day seven (late administration) after infection. In a separate set of experiments the effect of multiple dose administration of TIP versus saline was tested. Pulmonary edema was determined by the lung wet-to-dry (W/D) weight ratio and was assessed at different time-points after the administration of TIP. Secondary outcomes included clinical scores and lung cellular response.

**Results:**

TIP did not have an effect on pulmonary edema in different dose regimens at different time points during PVM infection. In addition, TIP administration did not affect clinical severity scores or lung cellular response.

**Conclusion:**

In this murine model of severe RSV infection TIP did not affect pulmonary edema nor course of disease.

## Background

Infection with the pneumovirus, respiratory syncytial virus (RSV) is an important cause of lower respiratory tract infection (LRTI) in young children [1]. The burden of RSV-induced LRTI is high and recently it was estimated that annually over 3.0 million young children with RSV-induced LRTI need to be admitted to the hospital worldwide. Despite years of research treatment for severe RSV-LRTI is limited to supportive care with oxygen and mechanical ventilation.

Histopathological studies of lungs of both animals and humans infected with respiratory syncytial virus show areas of pulmonary edema. Johnson *et al.* found mixtures of inflammatory cells, fibrin and edema in small airways of children with fatal RSV-LRTI [2]. In a study with baboons infected with human RSV massive pulmonary edema and vascular congestion was seen in lung tissue samples [3]. Several studies of mice infected with the pneumovirus pneumonia virus of mice (PVM), which is frequently used as a model for severe human RSV have shown that alveolar edema was present in lung tissue [4], [5]. Based on these studies mechanical obstruction of the small airways and alveoli by edema appears to play a significant role in RSV-induced respiratory failure. Although clinical studies on the consequences of pulmonary edema in RSV disease are lacking pulmonary edema is associated with prolonged respiratory failure and a higher mortality in other pulmonary conditions such as acute respiratory distress syndrome (ARDS) [6]–[8].

The increased accumulation of alveolar fluid may inactivate surfactant, increase surface tension, promote inflammation, accelerate further flooding and thus may contribute to the characteristic oxygenation anomalies during severe RSV-LRTI [9]. The accumulation of fluid in the infected lung is caused, in part, by injury of the alveolar- and capillary barrier leading to increased permeability and, in addition, by compromised alveolar fluid clearance (AFC) [10]. AFC depends on vectorial transport of salt and water across the alveolar epithelium in part through apically located epithelial sodium channels (ENaC), followed by extrusion into the lung interstitium via a basolaterally located Na,K-ATPase [11]. In vivo and in vitro studies have shown that RSV directly affects alveolar fluid clearance by decreasing active epithelial Na+ transport in different lung epithelial cells such as tracheal epithelial cells and Clara cells [12]–[14].

Recently, the lectin-like domain of tumor necrosis factor alpha (TNF-alpha) [15], has been recognized as an important regulator of alveolar fluid balance. The synthetic peptide AP301 that mimics the lectin-like domain of TNF-alpha (designated TIP) has shown to be able to improve AFC *in situ* and *ex vivo* in a flooded rat lung injury model and *in vivo* when applied intratracheally [16], [17] by both reducing vascular permeability and enhancing the absorption of excess alveolar fluid by up-regulating the sodium uptake. The same effect of TIP on AFC was found in an *in vivo* porcine broncho-alveolar lavage model of acute lung injury, in which nebulized TIP resulted in increased PaO_2_/FiO_2_ ratio and reduced extra vascular lung water index [18]. Vadász demonstrated that the effect of TIP was not limited to healthy lungs and found similar effects of TIP on alveolar liquid clearance in an *ex vivo* model of endo/exotoxin-induced lung injury [10]. Lucas *et al.* showed that in addition to its effect on pulmonary edema TIP also influenced inflammation by reducing neutrophil content and reactive oxygen species generation leading to improved lung function after lung transplantation in rats [19]. Together these studies suggest a promising role of TIP in treating pulmonary edema in several lung disease states and even the first safety studies in humans have been performed.

The aim of this study was to determine whether TIP reduces pulmonary edema in severe pneumovirus infection in mice. We used the pneumovirus PVM as a model of severe RSV infection in humans. PVM is capable of producing a form of respiratory disease in mice that is similar to severe RSV-LRTI in humans and has been shown to be a valuable alternative model of severe RSV infection to study the importance of virus-induced inflammatory responses in the development of severe respiratory virus disease [5], [20].

## Methods

### Viral stock preparation

PVM strain J3666 originally was orignially obtained from Dr. A. J. Easton (University of Warwick, UK) and was kept virulent by continuous passage in mice [21]. Clarified lung supernatants containing PVM were prepared by pooling the lungs of eight BALB/c mice infected with PVM. The pooled lungs were homogenized in 8 mL Isove's modified Dulbecco's medium (IMDM, Life Technologies, Gaithersburg, MD) and spun at 13,000×g for 5 min at 4°C. The supernatant was stored in individual aliquots in liquid nitrogen. Titers of PVM virus stocks were determined by isolation of RNA directly from virions in suspension with the RNeasy Mini Kit (Qiagen, Venlo, The Netherlands) and reverse transcribed to cDNA (high-capacity cDNA kit; Applied Biosystems, Bleiswijk, The Netherland). Copies of the PVM *sh* gene (GenBank AY573815) were detected in qPCR reactions with specific primers and TAMRA probe and normalized for copies of the houskeeping gene GAPDH as described before [22]. The virus titer in the aliquots was 12×10^4^ copies of PVM-*sh* per 10^9^ copies of *gapdh*/µl. On the day of each experiment, one aliquot was thawed and diluted (1∶1500) in RPMI medium (Invitrogen Ltd, Paisly, UK) for subsequent inoculation into the mice as described below.

### AP301

The cyclic peptide AP301 was a kind gift of APEPTICO, Vienna, Austria. Details of the synthesis have been described previously [23].

### Animal protocol

The animal protocols were approved by the Animal Care and Use Committee of the Academic Medical Center, University of Amsterdam, the Netherlands. Male C57BL/6J (C57) (Charles River laboratories, Someren, the Netherlands) aged 9- to 12-weeks-old received intratracheal instillations of 6×10^3^ copies of PVM in a total volume of 80 µl. Briefly, the mice were anesthetized with inhaled isoflurane and intubated endotracheally with a 22-gauge Insyte angiocath (BD, Madrid, Spain). Placement of the catheter in the trachea was verified by visualizing the movement of a 100 µl bubble of water in an open syringe in response to respiratory efforts as described before [22]. Following the instillations of PVM the mice were returned to their cages with free access to water and food. For the administration of TIP the mice were intubated again under isoflurane anesthesia and received TIP intratracheally in different doses and instillation volumes depending on the experiment. At different time-intervals after the TIP instillation the mice were euthanized with intraperitoneal ketamin 252 mg/kg, dexdomitor 0.4 mg/kg and atropin 1 mg/kg and exsanguinated by carotid artery ligation. The left lung was removed and weighed (wet weight). Bronchoalveolar lavage (BAL) was performed of the right lung by instilling three separate aliquots of 0.4 ml 0.9% NaCl containing 0.6 mM EDTA. One aliquot of BAL fluid (BALF) was processed immediately for cell counts and differentials. After the BAL, the right lung was inflated and fixed with formalin and embedded in paraffin for subsequent histological studies.

### Experimental design

The PVM-infected mice mice received TIP dissolved in normal saline intratracheally (dose, instillation volume and timing depending on the experiment as indicated) under isoflurane anesthesia. In all experiments control mice received saline without TIP in equal volumes.

### Measurements

#### Clinical score

The mice were monitored daily for clinical distress using weight loss and a specific score system as previously described [4]: 1 =  healthy, no signs of illness, 2 =  subtle ruffled fur, 3 =  evident ruffled fur with hunched posture, 4 =  evident lethargy with abnormal breathing pattern, 5 =  moribund, 6 =  death [modified from Cook et al. [24]. The end point for sacrifice used in this study was a score of 4 and/or loss of >20% of starting body weight.

#### Lung cellular response

The total number of BALF leukocytes was counted in a Bürker Bright line counting chamber. Cytospin preparations (20,000 cells per slide) were stained with the Diff-quick method (Fisher Scientific Company L.L.C., Kalamazoo, MI), and differential cell counts were obtained by counting 200 leukocytes using a standard light microscope.

#### Lung permeability

BALF total protein was measured with the bicinchoninic acid method (BCA assay, Pierce, Rockford, IL). The high molecular weight protein IgM was measured in BALF by ELISA (Bethyl Laboratories, West Chester, PA).

#### Pulmonary edema

We assessed pulmonary edema formation using the lung wet-to-dry (W/D) weight ratio [25]. The left lung was removed, quickly blotted dry and weighed (wet weight) on an analytical balance. Subsequently the lung was dried in a 65°C stove for seven days and weighed again (dry weight). In addition, the location of pulmonary edema was determined on hematoxylin and eosin (H&E) stained lung tissue sections.

### Statistical analysis

The data were analyzed using GraphPad Prism 4.0 software (GraphPad, San Diego, CA). Comparisons between multiple groups were performed using a two-way factorial analysis of variance (ANOVA), unless otherwise stated. Significance between groups was determined with the Bonferroni post hoc test. A p value of <0.05 was considered statistically significant. Data are reported as means ± standard error of the mean.

## Results

### Clinical response and pulmonary edema development during pneumovirus infection

**Figure 1:**
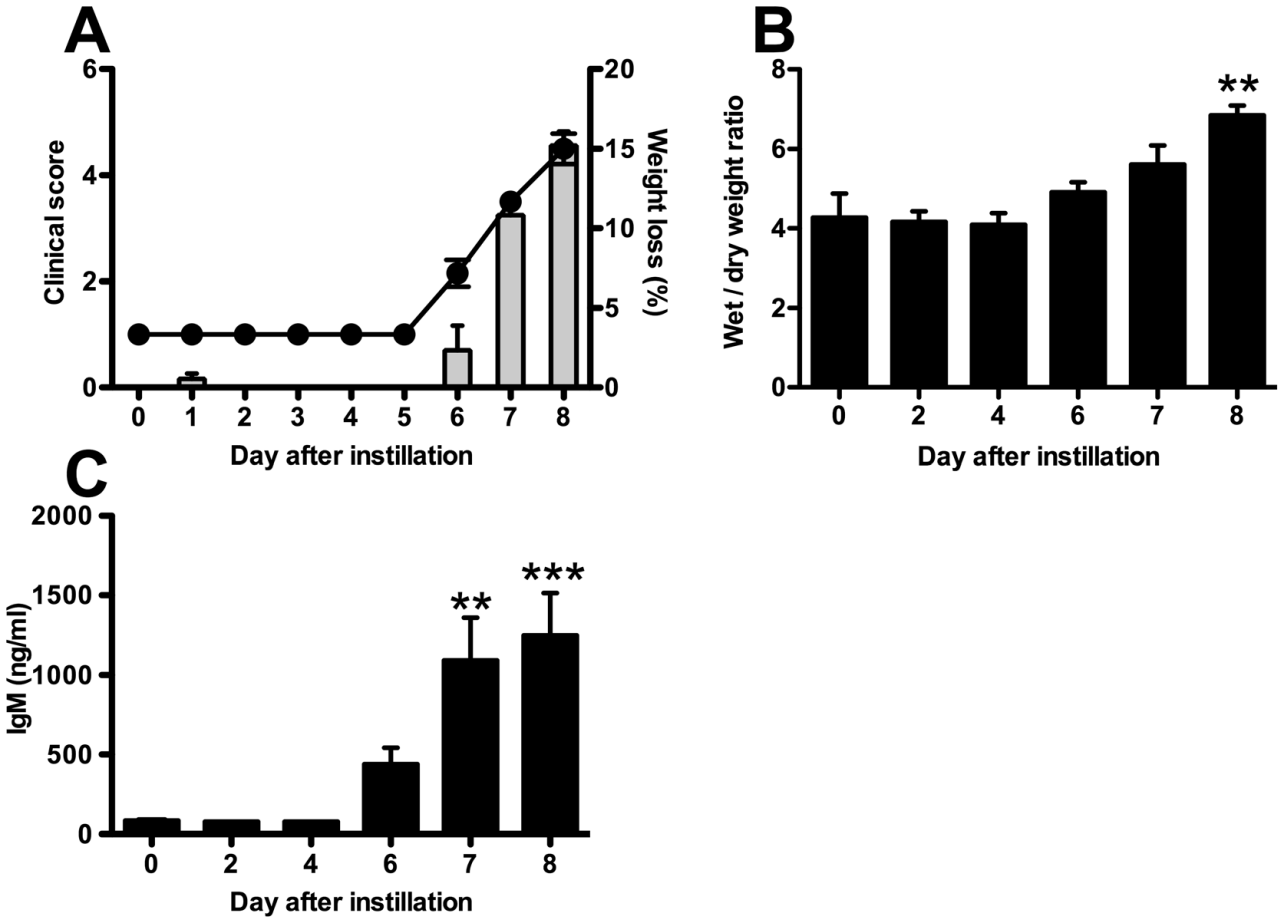
C57BL/6 mice intratracheally infected with 6×10^3^ copies of PVM develop clinical signs, increased alveolar permeability and pulmonary edema. *A*: mean clinical scores (left y-axis, line) and weight loss (right y-axis, bar) during infection with PVM. *B*: lung wet-to-dry weight ratio of C57BL/6 mice during PVM infection. *C*: IgM concentration in bronchoalveolar lavage fluid (BALF) of PVM-infected C57BL/5 mice. The graphs represent the mean of 4 mice at each time-point (± SEM). **p<0.01, ***p<0.001, as compared to uninfected mice (t = 0).

The first clinical signs of PVM disease in C57 mice appeared on day 6 after PVM infection and consisted of mild weight loss. The condition of the mice rapidly deteriorated and by day 8 the mice reached a clinical score of more than 4 and lost more than 10% of their body weight (Fig. 1*A*). Pulmonary edema was first detected on day 6 after infection, by an increase in lung W/D weight ratio of infected mice as compared to baseline (Fig. 1*B*), which reached significance on day 8 after infection (6.85±0.25 vs 4.27±0.61, p<0.01). The increase in pulmonary edema was paralleled by an increase in lung permeability as measured by an increase of the high molecular weight protein IgM in BALF of PVM-infected mice, which reached significance on day 7 and 8 after infection (day 0: 84.3±6.2 vs day 7: 1092.0±266.6, p<0.01 and vs day 8: 1248.0±266.7, p<0.001) (Fig. 1*C*). Thus, intratracheal instillation of PVM in C57 mice led to measurable clinical disease that was paralleled by an increase in pulmonary edema and lung permeability. Based on disease severity and the level of pulmonary edema on day eight we chose day 7 for our first intervention experiment with TIP.

### Intratracheal administration of TIP in different dose regimens did not affect pulmonary edema formation in PVM-infected mice

**Figure 2:**
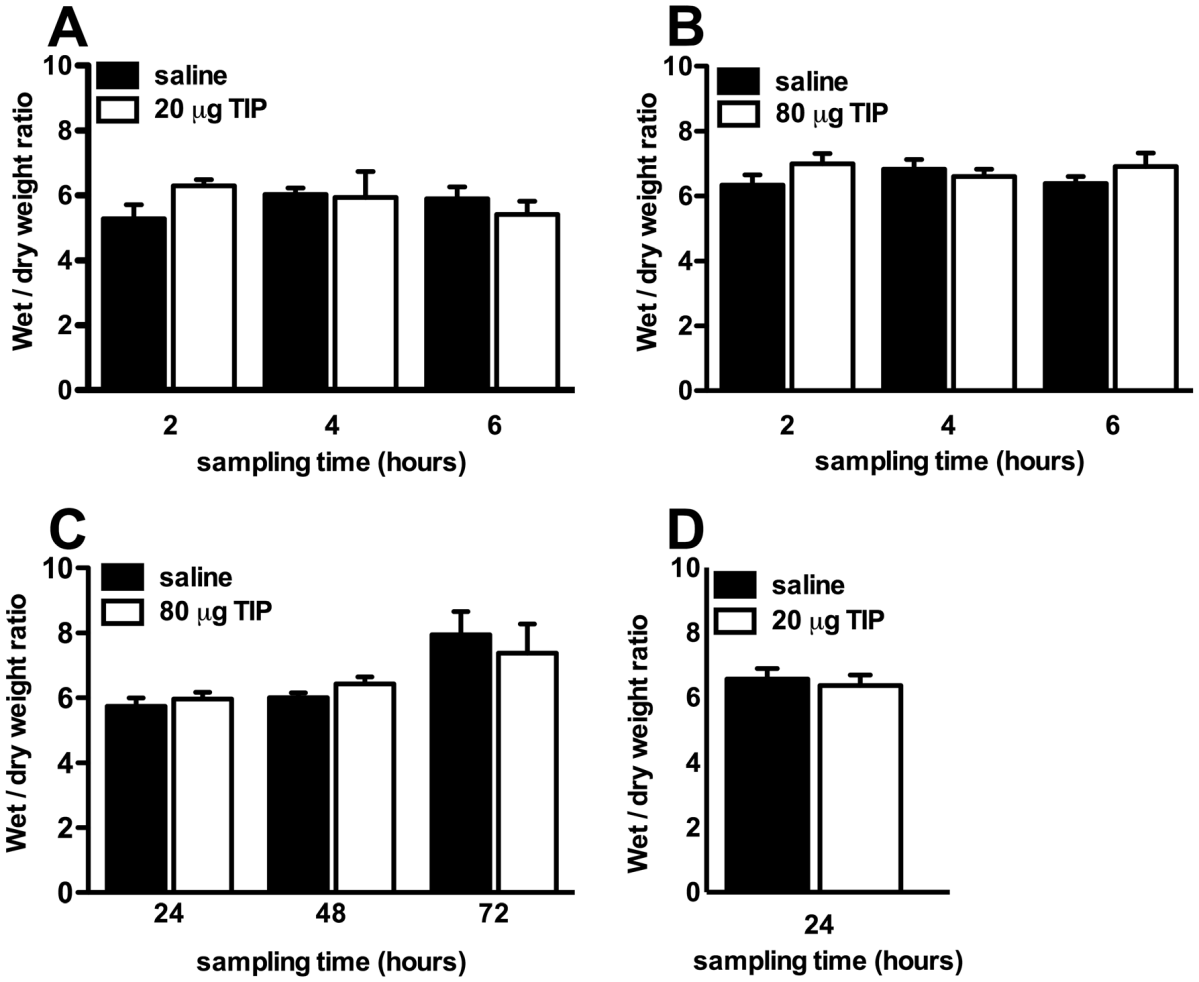
Intratracheal administration of TIP to PVM-infected C57BL/6 mice in different dose regimens and time schedules did not have an effect on pulmonary edema formation. *A*: Lung wet-to-dry weight ratio of the left lung of PVM-infected mice that received 20 µg of TIP in 30 µl saline or 30 µl saline on day seven after infection and were studied two, four or six hours later. *B*: Lung wet-to-dry weight ratio of the left lung of PVM-infected mice that received 80 µg of TIP in 60 µl saline or 60 µl saline (control) on day seven after infection and were studied two, four or six hours later. *C*: Lung wet-to-dry weight ratio of the left lung of PVM-infected mice that received 80 µg of TIP in 60 µl saline or 60 µl saline (control) on day 5 after infection and were studied 24, 48 and 72 hours later. *D*: Lung wet-to-dry weight ratio of the left lung of PVM-infected mice that received 20 µg TIP in 30 µl of saline or 30 µl saline on day zero, two, four and six after infection and were studied on day seven after infection. The graphs represent the mean of 4–8 mice at each time-point (± SEM).

Based on previous studies using TIP in mice infected intranasally with influenza strain A/PR8/34 (H. Fischer et al., unpublished data), we first studied the effect of 20 µg TIP in 30 µl saline. No difference in lung W/D weight ratio was found between PVM-infected mice that were treated with TIP and control animals at any time-point (2, 4, and 6 hours after installation of TIP (Fig. 2*A*). To determine if the lack of an effect of 20 µg TIP was due to either a low dose of TIP or a small instillation volume, we repeated the experiments with a higher dose of TIP (80 µg) in an instillation volume of 60 µl of saline. Again, no significant difference in lung W/D weight ratio between TIP treated mice and controls was found at any time point (Fig. 2*B*).

Because TIP was given late during the course of disease the benefits of TIP might be limited due to overwhelming disease. Therefore we determined the effect of early administration of TIP on pulmonary edema formation during PVM infection. Mice received 80 µg TIP in 60 µl saline on day 5 after PVM infection (before the increase of the lung W/D weight ratio, see Fig 1*B*). No significant difference was found in lung W/D weight ratio 24, 48 or 72 hours after instillation between PVM-infected mice that received TIP as compared to control mice (Fig. 2*C*).

Finally, in order to test if a multiple-dose regimen of TIP would prevent or reduce pulmonary edema formation during PVM infection, mice received intratracheal instillation of 20 µg TIP in 30 µl of saline on day 0, 2, 4 and 6 after PVM infection. The mice were euthanized 24 hours after the last TIP instillation. No difference was found in the lung W/D ratio between the mice that received TIP and control mice (Fig. 2*D*).

Effects of instillation volume (up to 60 µl) and isoflurane anesthesia on pulmonary edema were ruled out in separate experiments (data not shown).

**Figure 3:**
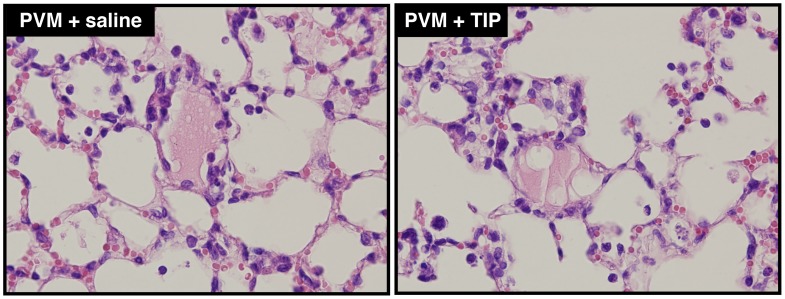
H&E staining of lung tissue from PVM-infected mice treated with TIP or saline. Representative H&E stained lung tissue sections from PVM-infected mice that were treated with 20 µg TIP in 30 µl saline (*right*) or 30 µl saline (*left*) on day seven after infection. The mice were studied two hours after the instillation of TIP or saline.

To determine if the lack of effect of TIP could be explained by the location of pulmonary edema, H&E stained lung tissue sections were examined. Similar to previous studies [4], [5], we observed protein-rich edema fluid in the alveoli in H&E-stained lung sections of PVM-infected mice, the same area where TIP is thought to affect ENaC-mediated Na^+^ transport. In line with our W/D ratio results we found no difference between TIP or saline treatment in these histological examinations (Fig. 3).

Thus, early or late intratracheal administration of 80 µg (single dose regimen) or 20 µg (single or multiple dose regimen) TIP in an instillation volume of 30–60 µl did not have an effect on pulmonary edema as measured by the lung W/D weight ratio of PVM-infected C57 mice.

### Administration of TIP during PVM infection did not have an effect on clinical disease progression or pulmonary inflammation

**Figure 4:**
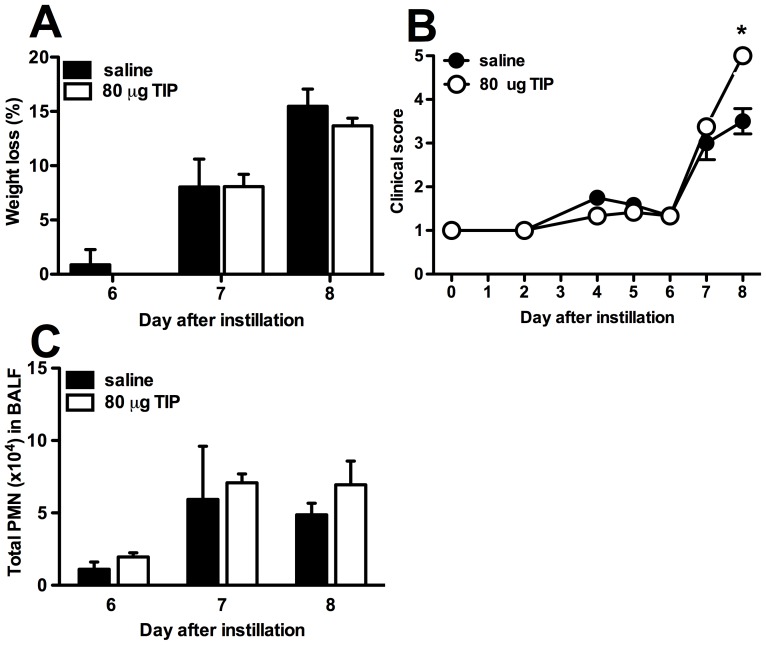
Single or multiple-dose regimen of TIP in PVM infection did not have an effect on weight loss, clinical score and lung cellular response. *A*: Change in body weight expressed as the percentage of the original weight of PVM-infected mice that received 80 µg of TIP in 60 µl saline (black bars) or saline (white bars) on day 5 after infection with PVM. *B*: Mean clinical score of PVM-infected mice that received 80 µg of TIP in 60 µl or saline on day 5 after infection with PVM, examined at daily intervals according to a standardized scoring system. *C*: Total polymorhonuclear neutrophils (PMN) measured in bronchoalveolar lavage fluid of PVM-infected mice that received 80 µg of TIP in 60 µl saline or saline on day 5 after infection with PVM. The graphs represent the mean of 4–6 mice at each time-point (± SEM). *p<0.05, **p<0.01, as compared to PVM-infected mice that received saline.

In the first experiment the mice were analyzed 2, 4 and 6 hours after the instillation of TIP on day 7 after PVM infection. No change in weight loss or clinical score was observed, as expected during this short time frame. In the second experiment the mice received TIP on day 5 after infection and were analyzed on day 6, 7 and 8. No difference in weight loss or clinical score was seen between TIP treated animals and controls on day 6 and 7 (Fig. 4A and B). An unexpected significantly higher clinical score was seen in the TIP-treated mice compared to controls on day 8 after PVM infection. However this was not paralleled by an increase in weight loss. Weight loss and clinical score were not different between PVM-infected mice that received multiple doses of TIP as compared to controls (data not shown).

Because Lucas et al. found a decreased number of alveolar neutrophils in transplanted lungs that were pretreated with TIP [19], we also measured the number of neutrophils in the BALF, but no significant difference between the mice that were treated with TIP and control mice was found in any of the experiments (Fig. 4C).

## Discussion

The aim of this study was to determine whether TIP reduces pulmonary edema in mice infected with PVM as a model for severe RSV infection in humans. We found that endotracheal administration of TIP in different dose regimens at different time points during the infection did not have an effect on pulmonary edema. In addition, TIP administration did not affect disease progression or lung cellular response.

Based on several histopathological studies [2]–[4], [26] on lungs of human and animals infected with the pneumoviruses RSV or PVM pulmonary edema is present and appears to be important in the disease pathogenesis. Pulmonary edema formation depends on disruption of both the pulmonary capillary endothelium and the alveolar epithelial barrier in combination with insufficient alveolar fluid clearance mechanisms. RSV can have potentially deleterious effects at each step of this process. The endothelial junction protein vascular endothelial (VE) cadherin is critical for maintenance of endothelial barrier integrity in lung microvessels. Leukocyte signaling cytokines and chemokines like interleukin (IL)-6 and IL-8 and destabilizing agonists such as VEGF are found in elevated levels in the airways of RSV-infected patients and it has been suggested that these mediators may contribute to edema formation by interrupting VE-cadherin bonds [27], [28]. In vivo mice studies and in vitro studies with respiratory epithelium have demonstrated that replicating RSV virus directly affects fluid clearance in the lungs in experimental models by inhibiting ENaC via uridine triphosphate (UTP) release in the airspace lining fluid [12], [13], [29], [30]. The mechanism behind the RSV induced UTP release is not known, but depends on active RSV replication. Various strategies to increase alveolar fluid clearance during RSV infection have been studied, but so far only treatment with leflunomide, an immunosuppressive agent that can decrease levels of UTP have shown promising effects in *in vivo* studies [13], [31]. Aerolized or intravenous beta-adrenergic agonist, which have shown to stimulate lung edema clearance in hyperoxic injured rat lungs by upregulating apical Na^+^ channels and basolateral N^+^/K^+^-ATPase treatment, failed to improve alveolar liquid clearance in RSV-infected mice [9]. In addition, in a trial in mechanically ventilated children with severe RSV infection investigating the effect of the immunomodulating drug dexamethasone, which besides anti-inflammatory effects also is known to increase α-ENaC mRNA expression *in vivo* and *vitro* [32], [33], no beneficial clinical effect was seen [34], [35].

This is the first *in vivo* study determining the effect of TIP on pulmonary edema in a viral infectious model. The lectin-like domain of TNF-α, mimicked by the 17 amino acid TIP peptide has been found to decrease hydrostatic pulmonary edema and endo/exotoxin-induced permeability edema in *in situ* and *ex vivo* animal studies [10], [16], [17]. TIP decreased vascular permeability and increased transepithelial Na^+^ transport and thus alveolar fluid clearance by upregulating both amiloride-sensitive Na^+^ channels and Na,K-ATPase in the epithelium in injured isolate rabbit lungs [10]. We hypothesized that TIP would decrease pulmonary edema in an *in vivo* murine model of pneumovirus induced lung injury. However, in contrast to the previous studies in other models and settings we did not find an effect of TIP in several regimens including various dosing and timing studies on pulmonary edema as measured by the lung W/D weight ratio or on clinical disease progression and lung cellular response in PVM-infected C57 mice.

There may be several explanations for the lack of an effect of TIP in our study. Firstly, the degree of endothelial and epithelial dysfunction differ between the PVM model and the experimental models in which TIP was studied before, eg hydrostatic and endo/exotoxin induced permeability edema. Studies using intra-alveolar and intravenous LPS demonstrated that endotoxin exposure increases lung vascular permeability, but does not affect the epithelial barrier or alveolar fluid transport [36], [37]. In contrast the PVM model is characterized by increased alveolar epithelial barrier permeability and decreased fluid transport as shown by an increased lung permeability as measured by an increase of the high molecular weight protein IgM in BALF. This might be an important difference, as interstitial edema resulting from disruption of the endothelial barrier will not lead to alveolar flooding unless the lung epithelial barrier, which is normally much tighter than the endothelium, also is compromised. This might affect the efficacy of TIP in the PVM model, as alveolar flooding might overwhelm any transport capacity of the epithelium. Secondly, Chen *et al.* found that RSV directly inhibits alveolar fluid clearance in human and rodent tracheal epithelial and human Clara cells through the release of UTP levels inhibiting Na^+^ transport [12]. The effect of RSV infection on AFC of the alveolar epithelial cells, the most important cell of oxygen transport in the lung, has to our best knowledge not been studied in detail. One could hypothesize that pneumovirus-induced UTP release is, among other mechanisms, also an important mechanism that influences AFC in alveolar epithelial cells and that as such up regulation of sodium uptake by TIP alone might not be sufficient to decrease pulmonary edema. Finally, ongoing viral replication and inflammation might have influenced the effectiveness of TIP in our model. Several studies investigating new treatment options in RSV disease have shown that dysregulated inflammation is an important process in the pathogenesis of pneumovirus disease [38]. Next studies should determine if TIP is more effective together with appropriate control of the ongoing inflammatory response or even with (new) antiviral agents.

The results of our study should be interpreted with caution. Pulmonary edema was assessed gravimetrically using the lung W/D weight ratio. Although, this method is common used method for pulmonary edema measurement it is sensitive for evaporative loss, regional heterogeneity or inclusion of blood in the wet lung weight [25], [39]. However, the W/D weight measurements were consistent and repeatable and were confirmed by the evaluation of the clinical response that did not show a difference between TIP-treated mice and controls.

## Conclusions

In conclusion we found no effect of TIP on pulmonary edema and clinical signs in a mouse model of severe RSV infection in humans. Further studies should focus on the effect of TIP on pulmonary edema in combination with anti-inflammatory agents and/or antiviral agents during pneumovirus disease.
